# Chronic Ouabain Prevents Na,K-ATPase Dysfunction and Targets AMPK and IL-6 in Disused Rat Soleus Muscle

**DOI:** 10.3390/ijms22083920

**Published:** 2021-04-10

**Authors:** Violetta V. Kravtsova, Inna I. Paramonova, Natalia A. Vilchinskaya, Maria V. Tishkova, Vladimir V. Matchkov, Boris S. Shenkman, Igor I. Krivoi

**Affiliations:** 1 Department of General Physiology, St. Petersburg State University, 199034 St. Petersburg, Russia; violettakravtsova@gmail.com (V.V.K.); mariiatiskova@gmail.com (M.V.T.); 2 Myology Laboratory, Institute of Biomedical Problems RAS, 123007 Moscow, Russia; inna199221@gmail.com (I.I.P.); vilchinskayanatalia@gmail.com (N.A.V.); bshenkman@mail.ru (B.S.S.); 3 Department of Biomedicine, MEMBRANES, Health, University of Aarhus, C 8000 Aarhus, Denmark; vvm@biomed.au.dk

**Keywords:** skeletal muscle, Na,K-ATPase isozymes, ouabain, resting membrane potential, hindlimb suspension, AMP-activated protein kinase

## Abstract

Sustained sarcolemma depolarization due to loss of the Na,K-ATPase function is characteristic for skeletal muscle motor dysfunction. Ouabain, a specific ligand of the Na,K-ATPase, has a circulating endogenous analogue. We hypothesized that the Na,K-ATPase targeted by the elevated level of circulating ouabain modulates skeletal muscle electrogenesis and prevents its disuse-induced disturbances. Isolated soleus muscles from rats intraperitoneally injected with ouabain alone or subsequently exposed to muscle disuse by 6-h hindlimb suspension (HS) were studied. Conventional electrophysiology, Western blotting, and confocal microscopy with cytochemistry were used. Acutely applied 10 nM ouabain hyperpolarized the membrane. However, a single injection of ouabain (1 µg/kg) prior HS was unable to prevent the HS-induced membrane depolarization. Chronic administration of ouabain for four days did not change the α1 and α2 Na,K-ATPase protein content, however it partially prevented the HS-induced loss of the Na,K-ATPase electrogenic activity and sarcolemma depolarization. These changes were associated with increased phosphorylation levels of AMP-activated protein kinase (AMPK), its substrate acetyl-CoA carboxylase and p70 protein, accompanied with increased mRNA expression of interleikin-6 (IL-6) and IL-6 receptor. Considering the role of AMPK in regulation of the Na,K-ATPase, we suggest an IL-6/AMPK contribution to prevent the effects of chronic ouabain under skeletal muscle disuse.

## 1. Introduction

The motor activity provided by skeletal muscles is obligatory for the normal function of the mammalian body, including humans [1,2,3]. Among the mechanisms for maintaining electrogenesis and contractile function of skeletal muscles, the activity of Na,K-ATPase plays a unique role. The Na,K-ATPase, discovered by Jens Christian Skou [4], is an integral protein that maintains Na^+^ and K^+^ transmembrane gradients due to their active transmembrane transport. Skeletal muscles contain the main pool of Na,K-ATPase in the body, which distribution density in the sarcolemma is in order of 1000/μm^2^ [2].

The Na,K-ATPase consists of catalytic and ion-transport α subunit and β subunit, mainly functioning as a chaperone [5,6,7]. In mammal cells, four isoforms of the α subunit and three isoforms of the β subunit are known. Various combinations of these subunits provide a wide molecular and functional diversity of the Na,K-ATPases [5,6,7,8,9]. Skeletal muscles co-express the α1 and α2 isoforms of Na,K-ATPase, whose physiological role and localization are important to define the contractile and metabolic function and is, therefore, an important object to study [10,11,12,13,14,15].

While the α1 Na,K-ATPase isozyme is relatively uniformly distributed in the sarcolemma, two main pools of the α2 isozyme are reported. The smaller α2 Na,K-ATPase pool is localized to the junctional (endplate) membrane, where it forms the multi-molecular complexes with nicotinic acetylcholine receptors (nAChRs), caveolin-3, and cholesterol to regulate the resting membrane potential (RMP) and thus, a safety factor for neuromuscular transmission [8,16,17]. The majority of extrajunctional α2 Na,K-ATPase isozyme is expressed in the interior transverse tubule membranes, where it prevents external K^+^ accumulation during muscle activity [13]. Although the α2 isozyme is predominantly expressed in adult muscles [10,11], the α1 Na,K-ATPase serves the main house-keeping role [16,18,19]. The α2 isozyme is more adaptive and is specifically regulated by skeletal muscle use [12,13,14,20].

Maintenance of the RMP level is essential for the excitability and normal function of skeletal muscle and even a small, but prolonged membrane depolarization can have dramatic consequences [21]. This depolarization inactivates sodium channels and suppresses membrane excitability, which is critical for neuromuscular transmission [17,22]. Notably, a sustained membrane depolarization is characteristic for both chronic [20,22,23] and relatively acute [14,24] motor dysfunction of different nature, predominantly due to an impairment of the Na,K-ATPase function. In the rodent model for reduced gravitational load, membrane depolarization develops within hours and precedes the overt skeletal muscle atrophy [14,24].

The extracellular loops of Na,K-ATPase α subunit form a specific binding site for cardiotonic steroids of plant and animal origin. These cardiotonic steroids, i.e., ouabain, digoxin, and marinobufagenin, have endogenous circulating analogs in the mammalian body [9,25,26,27]. Among these ligands, ouabain, which endogenous analogue synthesizes in the adrenal cortex and hypothalamus, is regarded as a hormone with the Na,K-ATPase as a receptor [9,25,26,27,28]. Endogenous ouabain circulates normally at subnanomolar concentration range, but its elevated level has been reported under physiological conditions such as exercise [29,30] and in a number of pathophysiological conditions [9,26,27,31,32]. Ouabain at high concentrations blocks the activity of Na,K-ATPase. However, at subnanomolar concentrations that correspond to the level of circulating endogenous analog, ouabain is shown to activate the Na,K-ATPase [33,34,35] and to hyperpolarize the sarcolemma [36]. Moreover, it has been shown that nanomolar concentrations of ouabain modulate interleikin-6 (IL-6) secretion and IL-6/STAT3 signaling pathways [37].

Increasing evidence indicates a broad therapeutic potential of circulating cardiotonic steroids, including ouabain [9,36,38,39,40,41]. The knowledge of molecular mechanisms behind the regulatory effects of circulating ouabain in skeletal muscle and their functional significance for muscle pathology could be useful for development of a new effective therapeutic strategy.

In this study, we hypothesized that the Na,K-ATPase is targeted by the elevated level of circulating ouabain to modulate the RMP and to prevent disuse-induced loss in skeletal muscle electrogenesis. We examined the ability of exogenous ouabain to modulate the Na,K-ATPase and electrogenesis of the rat soleus muscle under conditions simulating gravitational unloading in a standard hindlimb suspension (HS) model of disuse [42]. Conventional electrophysiology, Western blotting, and confocal microscopy with cytochemistry were used. We subjected rats to chronic injections of ouabain alone and in a combination with HS for 6 h. In some experiments, the effect of acute nanomolar ouabain administration was studied. We analyzed the RMP of muscle fibers at junctional and extrajunctional regions of the sarcolemma, electrogenic transport activity of the α1 and α2 Na,K-ATPase isozyme and their protein content, membrane localization of the α2 Na,K-ATPase and serum levels of ouabain and glucose. Considering the role of AMP-activated protein kinase (AMPK) in the regulation of the Na,K-ATPase in skeletal muscle [43] and its modulation in HS [44,45,46], the relative level of phosphorylation of AMPK, its substrate acetyl-CoA carboxylase (ACC), p70 protein, and mRNA expression of IL-6 and IL-6 receptor (IL-6R) also were assessed.

## 2. Results

### 2.1. An Acute Exposure to Nanomolar Ouabain Hyperpolarizes Sarcolemma via Enhanced Electrogenic Activity of the α2 Na,K-ATPase

**Figure 1:**
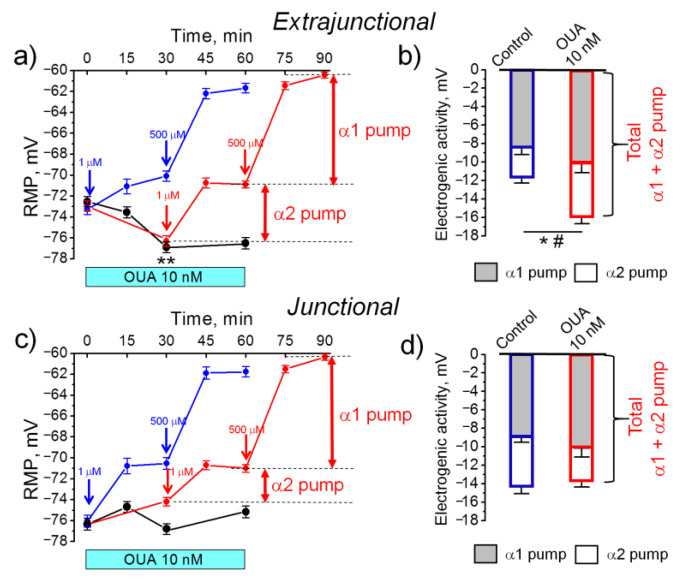
The effect of 10 nM ouabain on resting membrane potential (RMP) and electrogenic contribution of the α1 and α2 Na,K-ATPase in the sarcolemma of rat soleus muscles. The measurements were done in the (**a**,**b**) extrajunctional and (**c**,**d**) junctional regions of sarcolemma. (**a**,**c**) Black circles and lines show the RMP changes upon 10 nM ouabain incubation in bath solution at different time-points over 60 min experiment, as indicated. Red circles and lines show the RMP changes when bath ouabain concentration was increased from 10 nM to 1 µM and 500 µM (indicated by corresponding arrows) to estimate the electrogenic contribution of the α1 and α2 Na,K-ATPase (see also the Methods). Blue circles and lines demonstrate control measurements without pre-incubation with 10 nM ouabain, where the electrogenic contribution of the α1 and α2 Na,K-ATPase was estimated as above. (**b**,**d**) The estimated electrogenic contribution of the α1 and α2 Na,K-ATPase under control conditions and in the presence of 10 nM ouabain is shown in (**a**) and (**c**). Each data point is a mean value of membrane potentials measured in at least 100 fibers of 6–8 soleus muscles from 3–4 rats. One-way ANOVA. (**a**) ** *p* < 0.01—RMP compared to the corresponding initial value. (**b**) * *p* < 0.05—the α2 Na,K-ATPase contribution and # *p* < 0.05—total α1 and α2 Na,K-ATPase contribution compared with the control conditions.

We have previously reported that nanomolar ouabain hyperpolarizes the sarcolemma in rat diaphragm [36]. To assess whether this is a common phenomenon, we repeated similar measurements in rat soleus muscle. Incubation with ouabain (10 nM) in the bath solution hyperpolarized extrajunctional plasma membrane by −4.4 ± 0.7 mV (*p* < 0.01). The hyperpolarization achieved a steady state 30 min after ouabain incubation and remained stable within next 30 min (Figure 1a). Electrogenic contribution of the α2 Na,K-ATPase was estimated by its inhibition with 1 µM ouabain (see Methods). We found that the hyperpolarizing electrogenic contribution of the α2 Na,K-ATPase was increased from −3.2 ± 0.7 mV (measured in the control muscles without ouabain) to –5.4 ± 0.6 mV in the presence of 10 nM ouabain (*p* < 0.05) (Figure 1a,b). A further increase of ouabain to 500 µM, which inhibits the α1 Na,K-ATPase, showed that this isoenzyme electrogenic contribution was not significantly affected (Figure 1a,b). The total α1 and α2 Na,K-ATPase contribution increased from −11.6 ± 0.7 mV in control muscles up to −15.9 ± 0.4 mV (*p* < 0.05) in muscles pre-incubated with 10 nM ouabain (Figure 1b). Altogether, these measurements demonstrated that 10 nM ouabain hyperpolarized extrajunctional plasma membrane mostly via increased electrogenic activity of the Na,K-ATPase and that the α2 isoenzyme is preferentially modulated in this response (Figure 1b).

In the junctional sarcolemma region, 10 nM ouabain did not induce hyperpolarization (Figure 1c). While a tendency to reduction in electrogenic activity of the α2 Na,K-ATPase was observed, total α1 and α2 Na,K-ATPase contribution was not significantly affected (Figure 1c,d). These results are in accordance with previous report on differential regulation of the extrajunctional and junctional pools of the α2 Na,K-ATPase [14,36].

### 2.2. A Single Injection of Ouabain Does Not Prevent HS-Induced Disturbances in Muscle Electrogenesis

**Figure 2:**
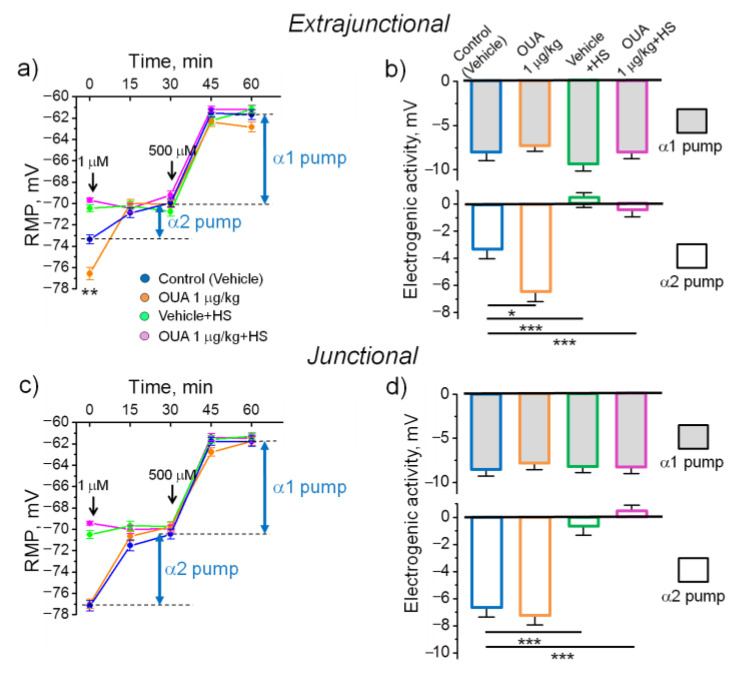
The effects of a single injection of ouabain prior to HS. The resting membrane potential (RMP) (**a**,**c**) and electrogenic contributions of the α1 and α2 Na,K-ATPase (**b**,**d**) in rat soleus muscles after 6 h of HS. The measurements were done in extrajunctional (**a**,**b**) and junctional (**c**,**d**) sarcolemma regions. Injection of either ouabain (1 µg/kg) or vehicle 0.9% NaCl was done immediately prior HS. The results were compared to 6-h measurements after vehicle (control) or 1 µg/kg ouabain injections (both without HS). The electrogenic contribution of the α2 and α1 Na,K-ATPase was estimated by administration of 1 µM or 500 µM ouabain (indicated by the corresponding arrows). Each data point corresponds to the averaged RMP measured in at least of 100 muscle fibers of 6–10 muscles from 3–5 rats. Two-way ANOVA followed by Tukey’s multiple comparisons test. (**a**) ** *p* < 0.01—RMP value compared to the corresponding control. (**b**,**d**) * *p* < 0.05, *** *p* < 0.001—compared as indicated by horizontal bars.

In these experiments, 6 h after a single injection of 1 µg/kg ouabain, extrajunctional membrane hyperpolarized from −73.4 ± 0.4 mV in control muscles to −76.6 ± 0.6 mV (*p* < 0.01) in ouabain-treated muscles. Respectively, the electrogenic contribution of α2 Na,K-ATPase was increased from −3.4 ± 0.6 mV up to −6.5 ± 0.7 mV (*p* < 0.05) (Figure 2a,b) and the total α1 and α2 Na,K-ATPase contributions were increased from −11.8 ± 0.6 mV to −13.7 ± 0.6 mV (*p* < 0.05). In the junctional membrane region, no significant difference between these groups was detected (Figure 2c,d).

In accordance with the previous report [14], 6 h of HS depolarized both extrajunctional and junctional membranes to approximately −70 mV due to decreased electrogenic contribution of the α2 Na,K-ATPase (Figure 2a–d). We questioned here, whether elevation of circulating ouabain could hyperpolarize muscle fibers and sustain their electrogenesis. However, a single injection of 1 µg/kg ouabain prior HS had no significant effect on the HS-induced depolarization (Figure 2a–d).

This suggests that the single injection of ouabain prior HS is ineffective to prevent HS-associated loss of the α2 Na,K-ATPase electrogenic contribution as well as membrane depolarization.

### 2.3. Chronic Ouabain Modulates the Na,K-ATPase Electrogenic Activity and Prevents the HS-Disturbed Electrogenesis

**Figure 3:**
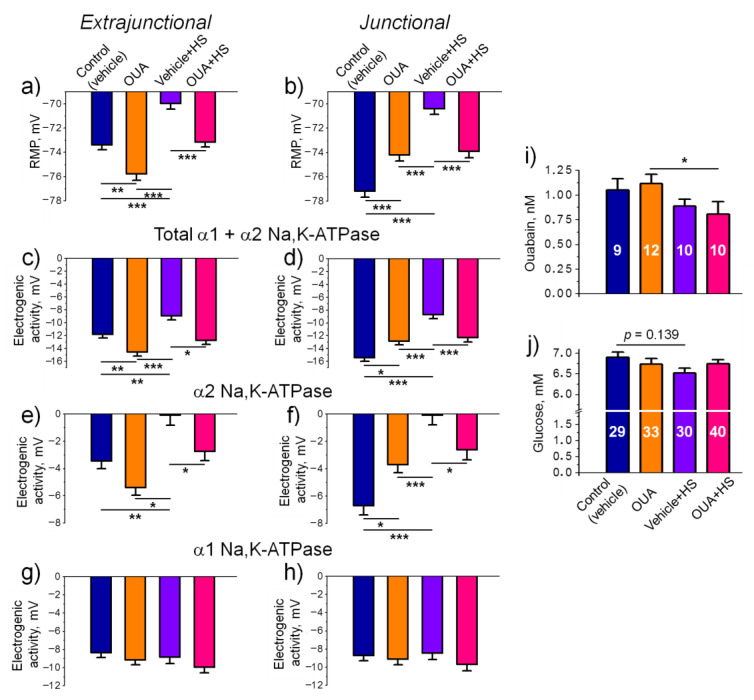
Changes in the electrogenesis of soleus muscles after 4-days pre-treatment with ouabain (1 μg/kg) followed by rat hindlimb suspension (HS) for 6 h. (**a**,**b**) Resting membrane potential (RMP). (**c**,**d**) Total electrogenic activity contributed by the α1 and α2 isozymes of Na,K-ATPase. (**e**,**f**) Electrogenic activity of the α2 isozyme of Na,K-ATPase. (**g**,**h**) Electrogenic activity of the α1 isozyme of Na,K-ATPase. The measurements in (**a**–**g**) extrajunctional and (**b**–**h**) junctional regions of the membrane, as indicated. (**i**,**j**) Serum level of ouabain and glucose, respectively (the number of rats is shown in the bars). Control and ouabain-treated (OUA) groups are represented by the rats, which received injections of vehicle (0.9% NaCl) or ouabain for 4 days, respectively. HS group contains the rats injected by 0.9% NaCl followed by HS for 6 h. OUA + HS group is a group of ouabain-injected rats that were exposed for the following 6-h HS. Each value is a result of measuring the RMP in at least 100 muscle fibers of 6–10 muscles from 3–5 rats. There was a significant interaction between HS and OUA effects for (**b**) *p* < 0.0001; *F* (1.614) = 42.15; (**d**) *p* < 0.0001; *F* (1.596) = 24.64 and (**f**) *p* < 0.0001; *F* (1.596) = 16.97. Two-way ANOVA followed by Tukey’s multiple comparisons test. * *p* < 0.05, ** *p* < 0.01, *** *p* < 0.001—compared as indicated by horizontal bars.

We injected rats intraperitoneally with ouabain (1 μg/kg) or 0.9% NaCl (vehicle) for 4 days and then exposed them to HS for 6 h. Similar to previous observations with the rat diaphragm muscles [36], chronic injections of ouabain caused hyperpolarization in the extrajunctional membrane region and depolarization in the junctional membrane region. However, this pre-treatment with chronic ouabain prevented the development of HS-induced depolarization in both regions of the sarcolemma (Figure 3a,b). The observed preventive effect was accompanied by corresponding increase in the total electrogenic activity contributed by the α1 and α2 isozymes of Na,K-ATPase, although predominant changes can be ascribed to the α2 isozyme activity (Figure 3c–h). These observations suggest that chronic injections of ouabain are required to prevent the HS-disturbed electrogenesis.

We found no significant changes in serum level of ouabain measured 30 h after last injection in rats, which were not exposed to HS, i.e., 24 h after injection and 6 more hours to match the timing with the HS group (Figure 3i). Importantly, chronic injections of ouabain followed by the HS caused a significant (*p* < 0.05) decrease in serum level of ouabain compared to the group that was treated with ouabain but did not exposed to the HS (Figure 3i). Blood glucose level was not significantly affected in all groups (Figure 3j).

### 2.4. Chronic Ouabain Reduces the α2 Na,K-ATPase Membrane Localization

**Figure 4:**
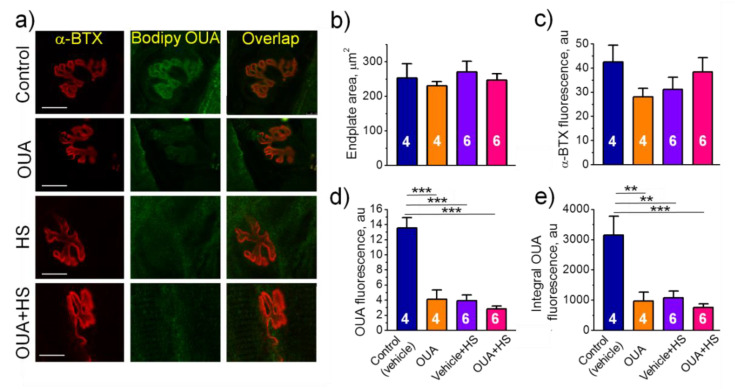
Characteristics of the α2 Na,K-ATPase localization in the rat soleus muscles. (**a**) Representative images of endplates dual-labeled with α-BTX (nAChRs, red channel) and BODIPY-conjugated ouabain (α2 Na,K-ATPase, green channel). Overlap—orange. Scale bars—10 μm. (**b**,**c**) Averaged area of endplates and fluorescence intensity (arbitrary units) from the nAChR staining, respectively. (**d**,**e**) Averaged fluorescence intensity and integral intensity over the entire area of endplates from the α2 Na,K-ATPase staining (arbitrary units). Control and ouabain-treated (OUA) groups are represented by the rats, which received injections of vehicle (0.9% NaCl) or ouabain for 4 days, respectively. HS group includes the rats injected by vehicle followed by HS for 6 h. OUA + HS group is a group of ouabain-injected rats that were exposed for the following 6 h HS. Each data point is a mean value measured in muscles from 4–6 rats, as indicated (17–50 endplates per group). There was a significant interaction between HS and OUA effects for (**d**) *p* = 0.0002; *F* (1.16) = 23.12); and (**e**) *p* = 0.0095; *F* (1.16) = 8.663. Two-way ANOVA followed by Tukey’s multiple comparisons test. ** *p* < 0.01, *** *p* < 0.001—compared as indicated by horizontal bars.

**Figure 5:**
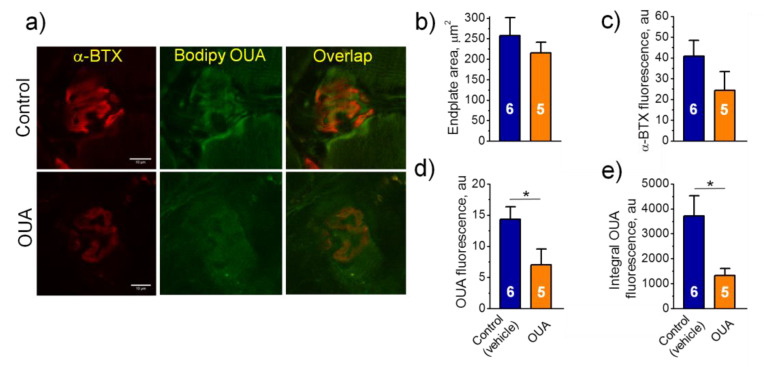
Characteristics of the α2 Na,K-ATPase localization in the rat diaphragm muscles. (**a**) Representative images of endplates dual-labeled with α-BTX (nAChRs, red channel) and BODIPY-conjugated ouabain (α2 Na,K-ATPase, green channel). Overlap—orange. Scale bars—10 μm. (**b**,**c**) Averaged area of endplates and fluorescence intensity (arbitrary units) from the nAChR staining, respectively. (**d**,**e**) Averaged fluorescence intensity and integral intensity over the entire area of the endplates from the α2 Na,K-ATPase staining (arbitrary units). Control and ouabain-treated (OUA) groups are represented by the rats, which received injections of vehicle (0.9% NaCl) or ouabain for 4 days, respectively. Each data point is a mean value measured in muscles from 5–6 rats, as indicated (21–19 endplates per group). One-way ANOVA. * *p* < 0.05—compared with the control (vehicle treated group).

To test the spatial localization of α2 Na,K-ATPase in the junctional region, we investigated images of soleus muscle endplates, which were dually labeled with fluorescent ligands of the Na,K-ATPase (BODIPY-conjugated ouabain) and the nAChR (rhodamine-conjugated α-BTX) (Figure 4a). BODIPY-conjugated ouabain was given at the concentration of 1 μM where it labels the α2 Na,K-ATPase only [16]. In all groups, the area of endplates as well as fluorescence intensity from the nAChRs did not significantly differ from the control group (Figure 4b,c). Chronic ouabain (1 μg/kg) alone or HS only significantly decreased fluorescence intensity as well as integral intensity over the entire area of endplates based on the α2 Na,K-ATPase labeling (Figure 4d,e). The observed effects correspond to decrease in the electrogenic activity of α2 Na,K-ATPase in the junctional membrane region (Figure 3f). The same effect of chronic ouabain was observed in diaphragm muscles (Figure 5), suggesting similar ouabain-dependent mechanism that does not dependent on muscle type. Pre-treatment with chronic ouabain did not prevent the HS-induced loss of the α2 Na,K-ATPase fluorescence (Figure 4d,e).

### 2.5. Chronic Ouabain Modulates Membrane Electrogenesis without Changes in the Na,K-ATPase Protein Content

**Figure 6:**
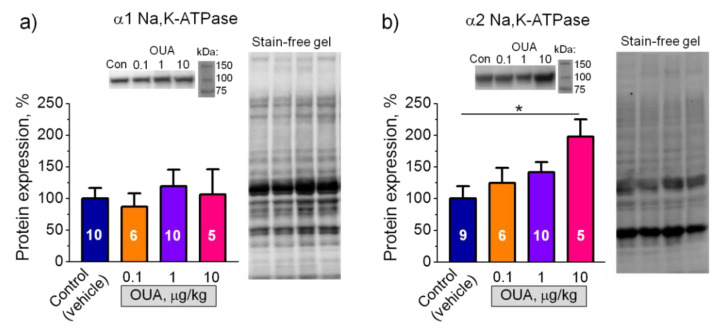
Effects of chronic exposure to ouabain on the protein content of α1 (**a**) and α2 (**b**) Na,K-ATPase in homogenate of rat soleus muscles. Rats were injected for 4 days with different doses (µg/kg) of ouabain as indicated. Control rats were injected with vehicle (0.9% NaCl). (**a**) and (**b**) Averaged Western blot results of the α1 and α2 isoform protein expression, respectively (the number of rats *n* = 5–10, as indicated); representative Western blots for semi-quantification of the Na,K-ATPase α1 and α2 isoforms are shown. Original images for Western blots using Stain-Free gels as a loading control are shown in Supplemental Materials. One-way ANOVA. * *p* < 0.05—compared with the control (vehicle treated group).

We further tested if chronic exposure to ouabain (0.1, 1, and 10 µg/kg) modulates the α1 and α2 Na,K-ATPase protein content measured in homogenates of soleus muscles (Figure S1). The α1 Na,K-ATPase protein level was unchanged at all ouabain doses (Figure 6a). The α2 Na,K-ATPase protein level was significantly (*p* < 0.05) increased only at 10 µg/kg ouabain (Figure 6b). The same effects of ouabain were observed earlier in the rat diaphragm muscle [36]. Thus, chronic ouabain at a dose of 1 µg/kg modulates the RMP and the Na,K-ATPase electrogenic activity without changes in the α1 and α2 Na,K-ATPase protein level.

### 2.6. Chronic Ouabain Pretreatment Modulates the Phosphorylation Level of AMPK and mRNA Expression of IL-6 under Disuse

**Figure 7:**
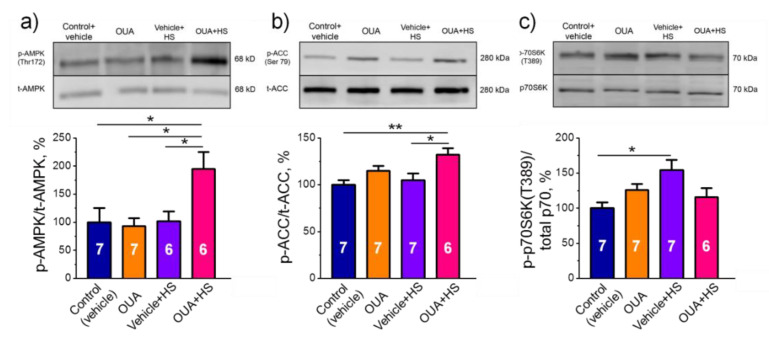
Pre-treatment with ouabain (1 μg/kg) for 4 days elevated the phosphorylation level of AMPK after 6-h HS. The relative phosphorylation levels of AMPK (**a**), ACC (**b**) and p70 (**c**) are shown. (**a**–**c**) Averaged immunoblots analysis (the number of rats *n* = 6–7, as indicated); the top panels show corresponding representative immunoblots. Original images for immunoblots are shown in Supplemental Materials. Control and ouabain-treated (OUA) groups are represented by the rats, which received injections of vehicle (0.9% NaCl) or ouabain for 4 days, respectively. HS group contains the rats injected by vehicle followed by HS for 6 h. OUA + HS group is a group of ouabain-injected rats that were exposed for the following 6 h HS. There was a significant interaction between HS and OUA effects for (**a**) *p* = 0.0373; *F* (1.22) = 4.915; and (**b**) *p* = 0.0093; *F* (1.23) = 8.071. Two-way ANOVA followed by Tukey’s multiple comparisons test. * *p* < 0.05, ** *p* < 0.01—compared as indicated by horizontal bars.

The relative phosphorylation level of AMPK, ACC and p70 was evaluated (Figure S2). Chronic exposure to ouabain alone or HS only did not change the relative phosphorylation levels of AMPK and ACC. However, a significant increase in the relative phosphorylation levels of AMPK and ACC were shown after HS, when rats were first chronically pre-treated with ouabain (Figure 7a,b). The relative phosphorylation level of p70 was significantly (*p* < 0.05) increased after HS, but was not affected in other groups (Figure 7c).

**Figure 8:**
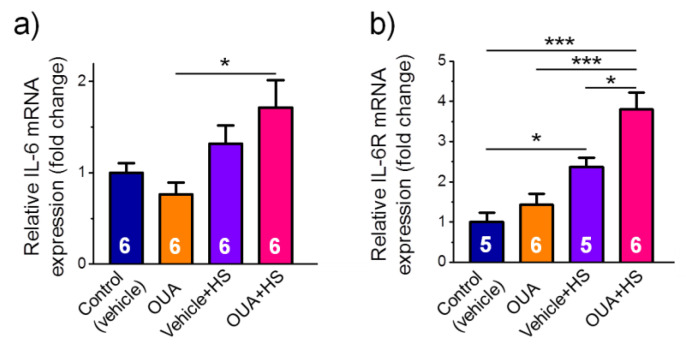
Pre-treatment with ouabain (1 μg/kg) for 4 days modulates mRNA expression of IL-6 (**a**) and IL-6R (**b**) after 6-h HS. Relative expression is shown. Control and ouabain-treated (OUA) groups are represented by the rats, which received injections of vehicle (0.9% NaCl) or ouabain for 4 days, respectively. HS group contains the rats injected by vehicle followed by HS for 6 h. OUA + HS group is a group of ouabain-injected rats that were exposed for the following 6 h HS. The number of rats *n* = 5–6, as indicated. Two-way ANOVA followed by Tukey’s multiple comparisons test. * *p* < 0.05, *** *p* < 0.001—compared as indicated by horizontal bars.

Although chronic exposure to ouabain (1 µg/kg) alone or HS only did not change IL-6 mRNA expression, a significant (*p* < 0.05) increase was seen after HS, when rats were first chronically pre-treated with ouabain (Figure 8a). The expression of IL-6R was significantly (*p* < 0.05) increased after HS but this increase was larger in the OUA + HS group (Figure 8b).

## 3. Discussion

Sustained membrane depolarization is characteristic for chronic skeletal muscle motor dysfunction seen in different animal models of myodystrophy [20,22,23]. This depolarization was shown to be associated with the Na,K-ATPase dysfunction and is among the earliest events that precede overt muscle atrophy [14,24]. The plasma level of endogenous ouabain increases in a variety of pathophysiological conditions [9,26,27,31,32] and under physical activity [29], suggesting that endogenous ouabain plays an important role in the regulation of skeletal muscle and cardiovascular system function. This ouabain action is, at least in part, proposed to be mediated via the ouabain-sensitive α2 Na,K-ATPase [30,47]. Notably, although ouabain is proposed to be a specific inhibitor of the Na,K-ATPase, its ability to activate the Na,K-ATPase at concentrations comparable to its endogenous level [33,34,35] and thus, to hyperpolarize the sarcolemma [36] was also shown. While the ability of nanomolar ouabain to activate intracellular signaling in cultured human skeletal muscle cells is well-documented [37,48], little is known about its role in the regulation of skeletal muscle function in vivo.

In this study, we tested the hypothesis that circulating ouabain can prevent the disuse-induced Na,K-ATPase dysfunction and sarcolemma depolarization in soleus muscle in rat HS model. The novelty of our findings is that:

(1) Although acute administration of 10 nM ouabain hyperpolarized the plasma membrane ex-vivo, a single injection of ouabain (1 µg/kg) prior the HS is unable to prevent the HS-induced membrane depolarization.
(2) Chronic administration of ouabain (1 µg/kg) alone for four days reduces the α2 Na,K-ATPase membrane localization without changes in the total level of α1 and α2 Na,K-ATPase proteins.
(3) Chronic administration of ouabain diminishes the HS-induced loss of the α2 Na,K-ATPase electrogenic activity and sarcolemma depolarization without changes in the α2 Na,K-ATPase localization in the junctional membrane.
(4) Chronic ouabain treatment increased the phosphorylation levels of AMPK and its substrate ACC accompanied with increased mRNA expression of IL-6 and IL-6R, suggesting the involvement IL-6/AMPK signaling pathways in preventive effects of ouabain.

Our findings suggest that the acute action of nanomolar ouabain concentration, which hyperpolarized the skeletal muscle ex-vivo, does not prevent the HS-induced membrane depolarization. In contrast, the HS-induced membrane depolarization was prevented by repeatable injections of ouabain for four days. This is in good agreement with a recent report that the modulatory effect of nanomolar ouabain on signaling pathways in cultured skeletal muscle cells was potentiated over time and was most potent after prolonged (12–24 h) treatment [37]. In our study, only the chronic administration of ouabain showed protective effects, suggesting the involvement of ouabain-triggered signaling pathways.

The signaling mechanism involved by the skeletal muscle motor dysfunction is rather complex and remains to be elucidated in details [45,49,50,51]. We found here two-fold increase in the relative phosphorylation level of AMPK in skeletal muscle from rats after chronic pre-treated with ouabain followed by the HS. Importantly, this was not the case for ouabain treatment alone or for the HS only. Among a variety of signaling molecules in skeletal muscle cells, AMPK is specifically targeted by disuse [44,45,46,50]. AMPK is an energy-sensing enzyme acting as a master-regulator for phenotypic determination and implicated in glucose, lipid and protein metabolism of skeletal muscle. It has been shown to play an important role in adaptations to physical exercise [52] at least in part, because AMPK signaling triggers downstream a transcriptional coactivator PGC-1α, a key protein for skeletal muscle adaptation to physical activity [53]. We suggest that reduced contractile activity of skeletal muscle in the HS leads to alterations in the intracellular ADP/ATP ratio leading to AMPK inactivation. Accordingly, pharmacological activation of AMPK by 5-aminoimidazole-4-carboxamide ribonucleotide (AICAR) was shown to prevent skeletal muscle pathology in different models of muscular atrophy [44,53,54].

Importantly, AMPK is also known as the Na,K-ATPase activator [43,55] and some data indicate that AMPK may regulate the Na,K-ATPase in skeletal muscle [43]. It was shown that activation of AMPK stabilizes the structure of rat soleus endplate and has a protective action on the RMP under conditions of the HS [56]. In this study, chronic ouabain alone significantly decreased fluorescence intensity of BODIPY-conjugated ouabain suggesting a decline in the α2 Na,K-ATPase membrane abundance. The mechanism behind it is unknown. We propose that chronic ouabain modulated the α2 Na,K-ATPase subunit trafficking between its intracellular pool and the sarcolemma. The possibility that ouabain modulates the α2 Na,K-ATPase turnover in the membrane also cannot be excluded. Notably, in the extrajunctional membrane region, the RMP and the Na,K-ATPase electrogenic activity under OUA and HS conditions were changed in opposing directions. In contrast, in the junctional membrane, synchronous reduce in muscle electrogenesis was observed under OUA and HS conditions and there was an interaction between OUA and HS groups. These observations correspond to decreased α2 Na,K-ATPase fluorescence in the junctional membrane region. Accordingly, an interaction between OUA and HS was also seen for fluorescence. From the other hand, chronic ouabain prevented the HS-induced loss in the Na,K-ATPase electrogenic activity. Considering the role of AMPK in regulation of the Na,K-ATPase and our findings, we suggest a crucial role of AMPK in this preventive action of ouabain.

Among the molecular mechanisms that contribute to adaptations to exercise, secretion of a variety of cytokines including IL-6 was previously recognized. The ability of nanomolar ouabain concentration to modulate IL-6 secretion and IL-6/STAT3 pathways in cultured human skeletal muscle cells was shown [37]. It was also previously demonstrated that muscle-derived IL-6 secretion could increase AMPK phosphorylation and activity [57,58]. Indeed, we observed in the present study a dramatic increase of IL-6 and IL-6R mRNA expression in the group of simultaneously ouabain-treated and hindlimb suspended rats. These findings allowed us to speculate that ouabain could increase AMPK phosphorylation during unloading via the Na,K-ATPase and IL-6 secretion. In the present study, we found an attenuation of the increase of phosphorylated p70S6k1 in ouabain-injected rats that were exposed to the HS. Accordingly, it was shown that p70S6k1 is phosphorylated at the early stage of unloading [59,60] and AMPK activation prevented it [60]. In the present study, the attenuation of p70S6k1 phosphorylation in ouabain-treated rats that were exposed to HS could be associated with the activated AMPK.

To enhance the concentration of circulating ouabain, intraperitoneal injections of ouabain at doses of 1–1.8 µg/kg are widely used [34,39,40]. In this study, rats were treated with ouabain at dose of 1 µg/kg for four days and this experimental protocol was shown doubled serum ouabain level from baseline 0.89 nM to 1.69 nM [34]. Previous pharmacokinetic studies demonstrated that plasma ouabain concentration after intravenous injection declines rapidly within first 7 h and then, displays a slow decay with a half-life of 18–24 h [61]. In our chronic experiments, serum ouabain level measured 30 h after last ouabain injection was not significantly increased compared to the baseline (1.05 ± 0.11 nM). However, injections of ouabain prior HS (OUA + HS group) caused a significant decrease in plasma ouabain level in comparison with the OUA group that was chronically treated with ouabain only, suggesting some interaction between these groups. We assume that extracellular potassium accumulated in the interior T-tubule [13] and synaptic clefts [62] of working muscles is decreased in disused hindlimb muscles. Notably, the majority of α2 isozyme is expressed in the T-tubule membrane [13] and only a smaller fraction of the α2 isozyme is localized at the junctional membrane [16]. As the external potassium antagonizes ouabain binding to the Na,K-ATPase [63], we suggest that ouabain binding might be increased in the disused hindlimb muscles. The skeletal muscles of adult albino rats account for 37–43% of the total body mass [64] and 16% of the total body mass account for two hindlimbs, where skeletal muscles make up to 71% of hindlimb mass [65]. Thus, we estimate that hindlimb skeletal muscles of rat account for approximately 30% of the total skeletal musculature and suggest that binding of ouabain in disused hindlimb muscles can significantly increase the clearance of ouabain from circulation. From another hand, because of increased binding, the functional effects of ouabain in the group with chronic injections of ouabain prior HS might be potentiated in comparison with other groups.

Oxidative stress might be another contributor to observed experimental results as it is an important molecular mechanism of muscle atrophy. Hindlimb unloading is known to increase oxidative stress [66,67], which may affect the activity of Na,K-ATPase in various cellular systems [68,69], including skeletal muscle [70]. Intraperitoneal injection of ouabain (1.8 µg/kg) is known to attenuate oxidative stress in lipopolysaccharide-induced neuroinflammation in rats [39]. Moreover, ouabain is suggested to play an important role in the control of NF-κB activation [71], where the α2 Na,K-ATPase/NF-κB pathway contributes to neuroinflammatory processes [72]. A link between the Na,K-ATPase and NF-κB shown in mdx mice [22] is of special interest and it would be interesting to examine, whether endogenous ouabain is important for the α2 Na,K-ATPase/NF-κB signaling in skeletal muscle pathology.

Altogether, we demonstrated a link between prolonged elevation of circulating ouabain and potentiation of IL-6/AMPK regulators and suggested their role in the prevention of functional decline of the Na,K-ATPase in disused muscles. We suggest that these findings are important for further studies of circulating ouabain in skeletal muscle and might have an important therapeutic implication.

## 4. Materials and Methods

### 4.1. Animals

Experiments were performed on male Wistar rats (180–230 g). Animals were housed in a temperature- and humidity-controlled room with food and water ad libitum. All procedures involving rats were performed in accordance with the recommendations for the Guide for the Care and Use of Laboratory Animals [73]. The experimental protocol met the requirements of the EU Directive 2010/63/EU for animal experiments and was approved by the Ethics Committee of St. Petersburg State University (issued 13 December 2017) and the Animal Experiments Inspectorate of the Danish Ministry of Environment and Food (issued 05 July 2016).

Rats were intraperitoneally injected with vehicle (0.9% NaCl) or 0.1, 1 and 10 µg/kg body weight ouabain once daily for 4 days, as described previously [34,36]. Twenty-four hours after the last ouabain (1 µg/kg) injection, rats were subjected to the HS that widely used as an animal model of disuse leading to progressive atrophy of postural skeletal muscles. The rats were subjected to the HS individually in custom-made cages for 6 h, as described previously [42]. Control animals were not suspended. Thirty hours after last injection of vehicle or ouabain (twenty-four hours after injection +6 h to match timing with the HS) soleus muscles were isolated. Before tissue harvest, the animals were euthanized by intraperitoneal injection of a tribromoethanol overdose (750 mg/kg) followed by cervical dislocation.

Freshly isolated soleus muscles were immediately used for electrophysiological experiments or confocal microscopy imaging. For later biochemical assays, some muscles were snap-frozen in liquid nitrogen and then stored at −80 °C. In some experiments, a single injection of 1 µg/kg ouabain prior 6 h of the HS was performed.

In a separate set of experiments, ouabain at 10 nM concentration was acutely added to isolated intact soleus muscles obtained from non-treated rats.

The serum level of ouabain was estimated using ELISA Kit for Ouabain (Cloud-Clone corp., Katy, TX, USA). The blood glucose level was measured with electronic blood glucose meter (Accu-Chek Active, Roche Diabetes Care GmbH, Mannheim, Germany).

### 4.2. Membrane Potential Recording

The isolated muscle with nerve stump was placed in a chamber and continuously perfused with physiological solution containing (in mM): NaCl, 137; KCl, 5; CaCl_2_, 2; MgCl_2_, 2; NaHCO_3_, 24; NaH_2_PO_4_, 1; glucose, 11; pH 7.4. The solution was continuously gassed with 95% O_2_ and 5% CO_2_ and maintained at 28 °C. The RMP was recorded from the surface fibers using intracellular glass microelectrodes. The RMP recordings were made in the extrajunctional membrane regions within ~2 mm from visually identified terminal branches of the nerve, or directly near the nerve terminals, as described previously [16,74]. In each muscle, RMPs were recorded from ~25 different fibers for each (junctional and extrajunctional) membrane region over a total time of about 5–10 min.

### 4.3. Measurement of the Na,K-ATPase Electrogenic Activity in Intact Muscle

The Na,K-ATPase electrogenic transport was determined in intact muscle by measuring ouabain-sensitive changes in the RMP. These changes are generated by electrogenic Na,K-ATPase transport. This is a previously characterized sensitive real-time assay to assess the Na,K-ATPase activity in intact skeletal muscle [16,18,22]. This method is based on more than 100-fold difference in affinities of the rodent α1 and α2 Na,K-ATPase isoforms for ouabain. Thus, in rat skeletal muscle, 1 μM ouabain inhibits the α2 isoform without affecting the α1 isoform, whereas 500 μM ouabain completely inhibits both isoforms [16,18]. The electrogenic contribution of α2 isozyme was computed as the difference in mean RMP before and 30 min after the incubation with 1 µM ouabain. The electrogenic contribution of α1 isozyme was estimated as the difference in RMP with 1 µM ouabain and after 30 min incubation with 500 µM ouabain.

### 4.4. Confocal Microscopy Imaging

To identify the endplate membrane region, tetramethylrhodamine-α-bungarotoxin (α-BTX, Biotium, Fremont, CA, USA), a fluorescent-labeled specific ligand of the nAChRs was used. For selective imaging of the α2 Na,K-ATPase, a freshly isolated muscle was incubated for 15 min with physiological saline containing fluorescent-labeled specific ligands of the Na,K-ATPase (BODIPY-conjugated ouabain, 1 μM, a concentration that preferably affects the α2 Na,K-ATPase membrane pulls [16] and the nAChRs (rhodamine-conjugated α-BTX, 1 μM). The physiological solution was bubbled with 95% O_2_ and 5% CO_2_ and maintained at room temperature. Superficial regions of the muscle were imaged with a ×63, 1.3 NA objective using a Leica TCS SP5 confocal system configured for concurrent viewing of rhodamine and BODIPY fluorescence, as described previously [16].

Analysis of endplate fluorescence was performed in the region defined by α-BTX staining. The fluorescence intensity from the nAChRs and the α2 Na,K-ATPase was determined from using ImageJ software. Each distinct region (fragment) of the nAChRs labelling was outlined by hand using “freehand” tool. The resulting outlines (masks) were then used to determine the localization of α2 Na,K-ATPase fluorescence at the junctional (endplate) membrane region. The fluorescence intensity (arbitrary units) was defined as the ratio of fluorescence intensity to the area of endplate. The mean endplate area and fluorescence intensities were assessed for each rat and then averaged.

### 4.5. Western Blot Assays

Muscle was lysed in lysis buffer (in mM: Tris-HCl 10, sucrose 250, EDTA 1, EGTA 1, Triton X-100 2%, pH 7.4; and 1 tablet protease inhibitor per 10 mL). The homogenate was centrifuged at 10,000× *g*. Total protein concentrations in the supernatants were measured using BCA Protein Assay Kit (Thermo Fisher Scientific, Waltham, MA, USA). Ten micrograms of total protein diluted in Laemmli sample buffer (Bio-Rad, Hercules, CA, USA) were loaded to 4–20% precast polyacrylamide stain-free gels (CriterionTM TGX Stain-freeTM precast gel, BioRad, Hercules, CA, USA). Total protein load was detected on the stain-free gels using UV-light in imaging system (c600, Azur Biosystems Inc., Dublin, CA, USA). The proteins were electrotransferred to nitrocellulose membranes that were then blocked by an incubation in 5% bovine serum albumin and 5% nonfat dry milk in PBS with 0.5% *v*/*v* Tween 20 (PBS-T). The membranes were incubated overnight at 5 °C with either α1 isoform Na,K-pump antibody (monoclonal, HPR-conjugated, 1:2000; Novus Biologicals Inc., Centennial, CO, USA) or with antibody against the α2 isoform (1:2000, Merck Millipore, Burlington, MA, USA). After intensive washing, the membranes were incubated with horseradish-peroxidase (HRP)-conjugated secondary antibody (1:4000; Dako Agilent, Santa Clara, CA, USA) for 1 h in PBS-T. Excess antibody was removed by washing, and bound antibody was detected by an enhanced chemiluminiscence kit (ECL, Amersham, Little Chalfont, UK). Detected protein was normalized using the ImageJ program (NIH, Bethesda, MD, USA) as a ratio to total protein load measured for the same probe.

### 4.6. Cytoplasmic Extracts Preparation and Immunoblots

Cytoplasmic extracts were prepared from 50 mg of soleus muscle using NE-PER Nuclear and Cytoplasmic Extraction Reagents (Thermo Scientific, Rockford, IL, USA). Complete Protease Inhibitor Cocktail (Santa-Cruz, CA, USA), Phosphatase Inhibitor Cocktail B (Santa Cruz, CA, USA), PMSF (1 mM), aprotinin (10 μg/mL), leupeptin (10 μg/mL), and pepstatin A (10 μg/mL) were used to maintain extract integrity and function.

The protein content of all samples was quantified twice using a Quick Start Bradford Protein Assay (Bio-Rad Laboratories) in order to calculate the optimal sample value for electrophoretic gel. The supernatant fluid was diluted with 2× sample buffer (5.4 mM Tris-HCl, pH 6.8, 4% SDS, 20% glycerol, 10% β-mercaptoethanol, 0.02% bromphenol blue) and stored at −85 °C for immunoblot procedures.

For each protein analyzed, conditions of electrophoresis and Western blotting were optimized, according to the protein molecular weight and quantity in the lysate. Electrophoresis was carried out in the 10% separating polyacrylamide gel (0.2% methylene-bisacrylamide, 0.1% SDS, 375 mM Tris-HCl, pH 8.8, 0.05% ammonium persulfate, 0.1% TEMED) and in the 5% concentrating polyacrylamide gel (0.2% methylene-bisacrylamide, 0.1% SDS, 125 mM Tris-HCl, pH 6.8, 0.05% ammonium persulfate, 0.1% TEMED). The cathode (192 mM Tris-glycine, pH 8.6, 0.1% SDS) and anode (25 mM Tris-HCl, pH 8.6) buffers were used. For each sample, 20 μg of cytoplasmic protein was loaded. The samples of each group were loaded on the gel together with matching control samples. Electrophoresis was carried out at 17 mA/gel in a mini system (Bio-Rad Laboratories) at room temperature.

Electrotransfer of the proteins was carried out in buffer (25 mM Tris, pH 8.3, 192 mM glycine, 20% ethanol, 0.04% SDS) to nitrocellulose membrane at 100 V and 4 °C in the Mini Trans-Blot system (Bio-Rad) for 120 min. The membranes were blocked in 5% non-fat dry milk solution (Bio-Rad) in PBST (phosphate-buffered saline pH 7.4, 0.1% Tween 20) for 1 h at room temperature. To reveal protein bands, the following primary polyclonal antibodies were used: Phosphorylated Thr 183/172 AMPKα1/2 (1:1000, ABM, San Francisco, CA, USA, #Y408289), AMPKα (1:1000, Cell Signaling Technology, Danvers, MA, USA, #2532), phosphorylated Ser 79 ACC (1:1000, Cell Signaling Technology, Danvers, MA, USA, #3661), total ACC (1:2000, Cell Signaling Technology, Danvers, MA, USA, #2072), phosphorylated Thr 389 p70S6K (1:2000, Cell Signaling Technology, Danvers, MA, USA, #11759), total p70s6k (1:1000, Cell Signaling Technology, Danvers, MA, USA, #9202), GAPDH (1:10,000, Applied Biological Materials Inc., Richmond, British Columbia, Canada, #G041). All the primary antibodies were used for overnight incubation at 4 °C. The secondary HRP-conjugated antibodies (goat-anti-rabbit, Santa Cruz, CA, USA, 1: 30,000, goat-anti-mouse, Santa Cruz, CA, USA, 1: 25,000) were used for 1-h incubation at room temperature. The blots were washed 3 times, 10 min each, in PBST. Then the blots were revealed using the ImmunStar TM Substrate Kit (Bio-Rad Laboratories, Hercules, CA, USA) and the C-DiGit Blot Scanner (LI-COR Biotechnology, Lincoln, NE, USA). Protein bands were analyzed using the Image Studio Digits Ver. 4.0 software. All image densities were measured in linear range of scanner detection. The optical absorption of the control group band on analytical membrane was taken as 100%, while the optical absorption of other groups was compared to the control group bands localized on the same membrane. The blots on which phosphorylated proteins were detected were stripped with Restore Western Blot Stripping Buffer (Thermo Scientific) and then, re-probed with total protein antibodies overnight at 4 °C. Then, the blots were incubated with HRP-conjugated goat-anti-rabbit secondary antibody and visualized as described above. It was controlled that phosphorylated proteins, goat-anti-rabbit-HRP complexes, were washed out completely from the blots. The blots were washed 3 × 10 min at room temperature with PBST after incubations with the antibodies and Restore Western Blot Stripping Buffer. Phosphorylated proteins were normalized to total proteins content. Ponceau S staining of the membranes was utilized to ensure equal loading of the extracts (not shown). All Western blots were repeated at least three times.

### 4.7. RT-qPCR Analysis

Reverse transcription was performed by incubation of 0.5 μg of RNA, random hexamers d(N)6, dNTPs, RNase inhibitor and MMLV reverse transcriptase for 60 min at 37 °C. Samples to be compared were run under similar conditions (template amounts, duration of PCR cycles). The annealing temperature was based on the PCR primers optimal annealing temperature. PCR primers used for RNA analysis are shown in Table 1. The amplification was real time monitored using SYBR Green I dye and the iQ5 Multicolor Real-Time PCR Detection System (Bio-Rad Laboratories, USA). To confirm the amplification specificity, the PCR products from each primer pair were subjected to a melting curve analysis and sequencing of the products was provided at least once. Relative quantification was performed based on the threshold cycle (CT value) for each of the PCR samples [75]. RPL19 was tested and chosen for the normalization of all quantitative PCR analysis in the current study.

### 4.8. Materials

Ouabain and other chemicals were purposed from Sigma-Aldrich.

### 4.9. Statistics

Data are given as mean ± SEM. The statistical significance of the difference between means was evaluated using one-way or two-way ANOVA followed by Tukey’s multiple comparisons test where appropriate. Statistical analysis was performed using GraphPad Prism 8 software (GraphPad; San Diego, CA, USA). A probability value of *p* < 0.05 was considered statistically significant.

## Figures and Tables

**Table 1:** Primers used for RT-qPCR analysis.

Gene Description	Forward Primer Reverse Primer	GeneBank
*Rpl 19*	5′-GTACCCTTCCTCTTCCCTATGC-3′ 5′-CAATGCCAACTCTCGTCAACAG-3′	NM_031103.1
*Interleukin-6*	5′-CCGGAGAGGAGACTTCACAG-3′ 5′-ACAGTGCATCATCGCTGTTC-3′	NM_012589.2
*Interleukin-6 receptor*	5′-TCACAGAGCAGAGAATGGACT-3′ 5′-GTATGGCTGATACCACAAGGT-3′	NM_017020.3

## Data Availability

The data that support the findings of this study are available from the corresponding author upon reasonable request.

## Supplementary Materials

The following are available online at https://www.mdpi.com/article/10.3390/ijms22083920/s1, Figure S1: Original Images for Figure 6, Figure S2: Original Images for Figure 7.
