# Supplementary material for: Chronic Ouabain Prevents Na,K-ATPase Dysfunction and Targets AMPK and IL-6 in Disused Rat Soleus Muscle
Source: Int J Mol Sci. 2021 Apr 10;22(8):3920. doi: 10.3390/ijms22083920 (PMC8069997; doi:10.3390/ijms22083920)
Supplement: Supplementary file 1 [file ijms-22-03920-s001.pdf]

Figure S1. Original Images for Figure 6.

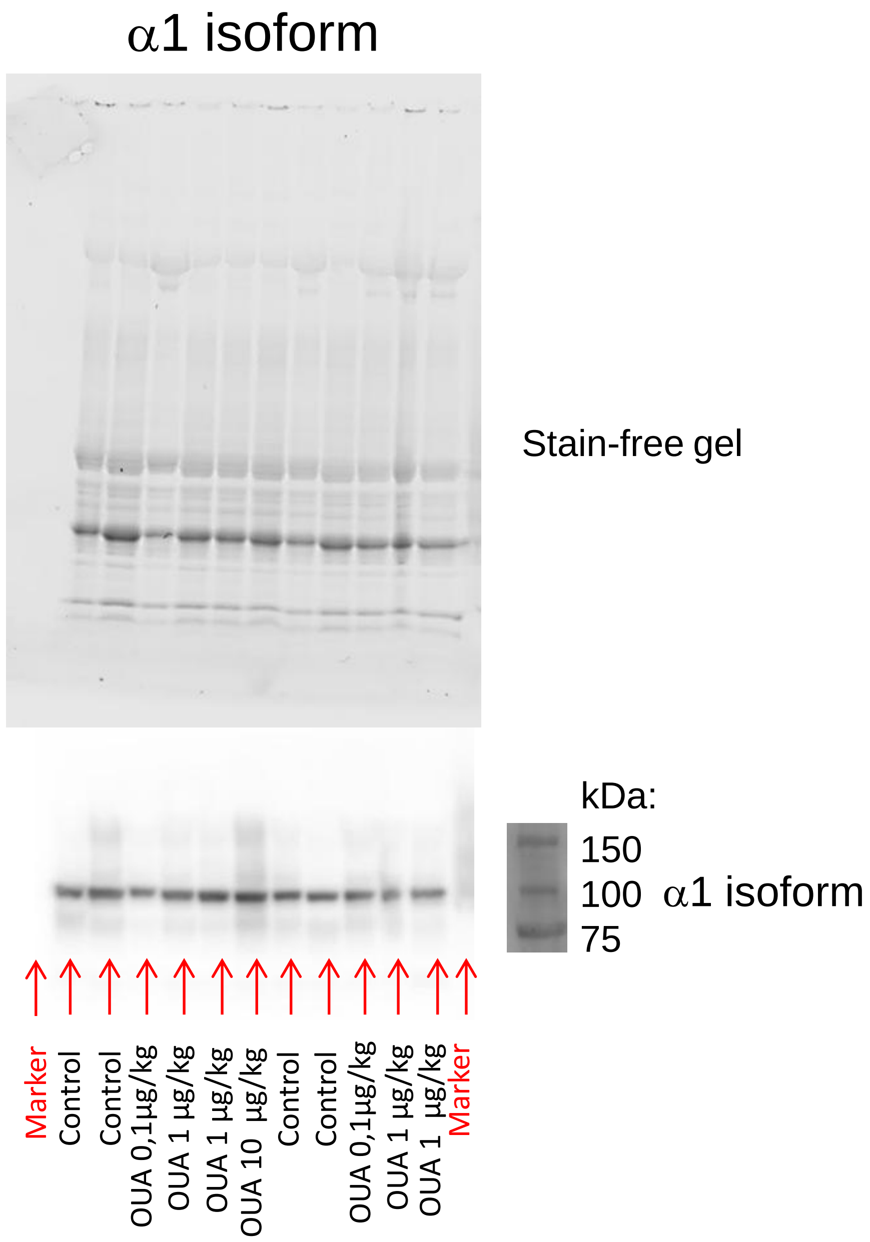

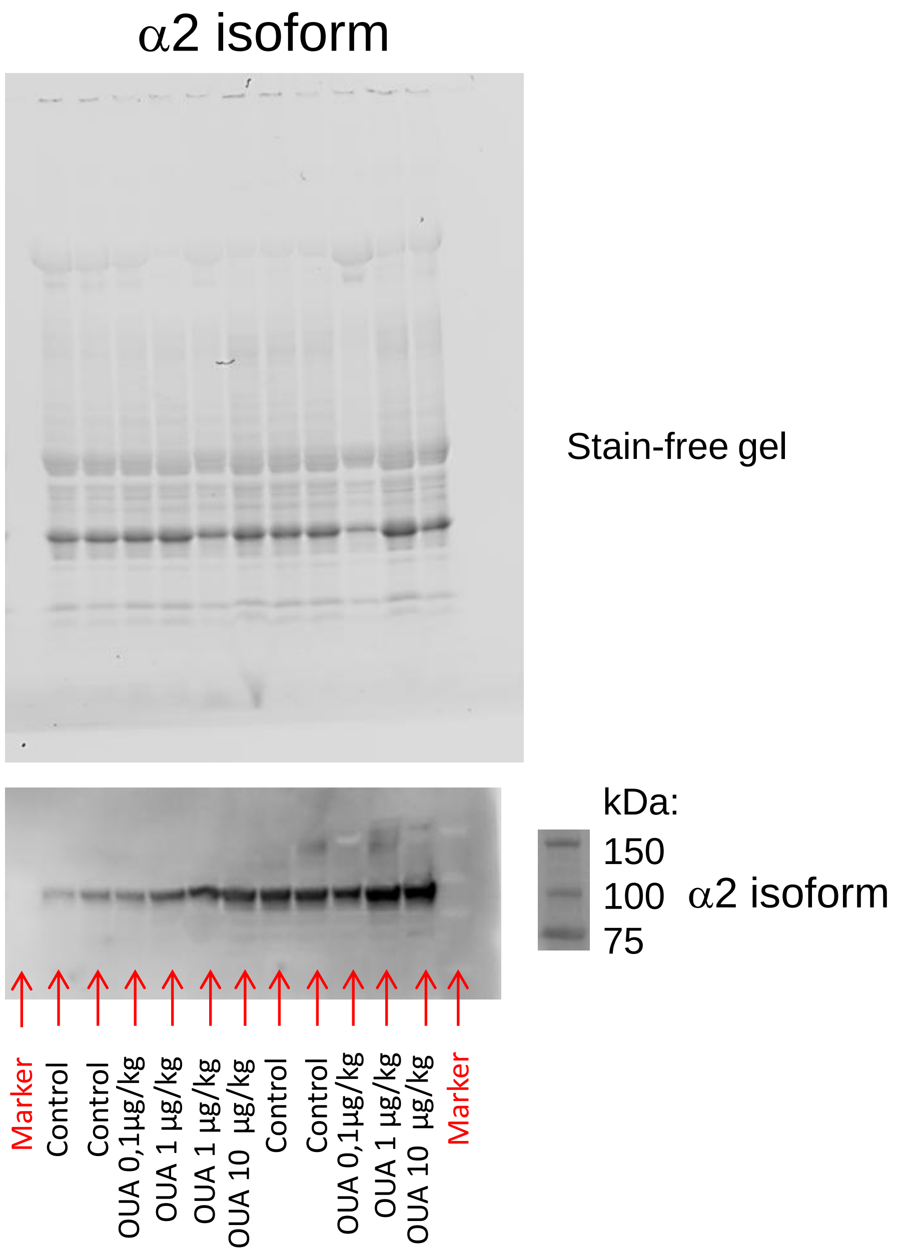

Figure S2. Original Images for Figure 7.

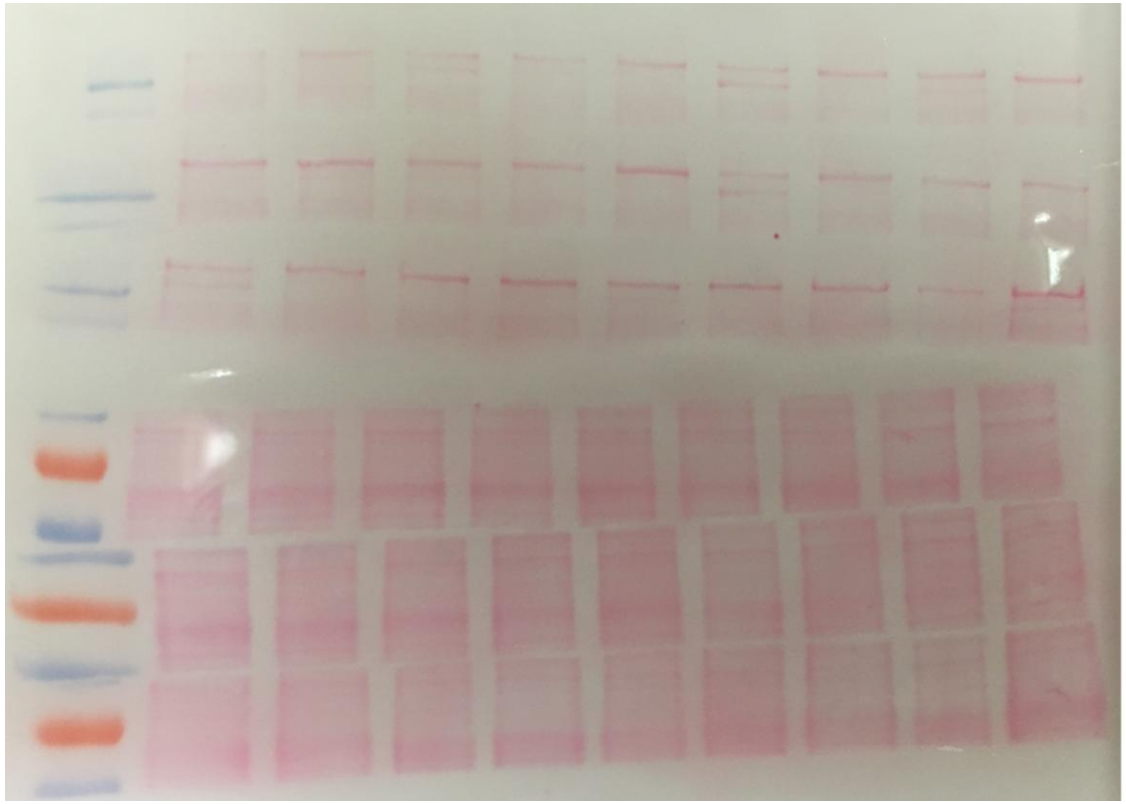

Ponceau

ACC

Ponceau

AMPK

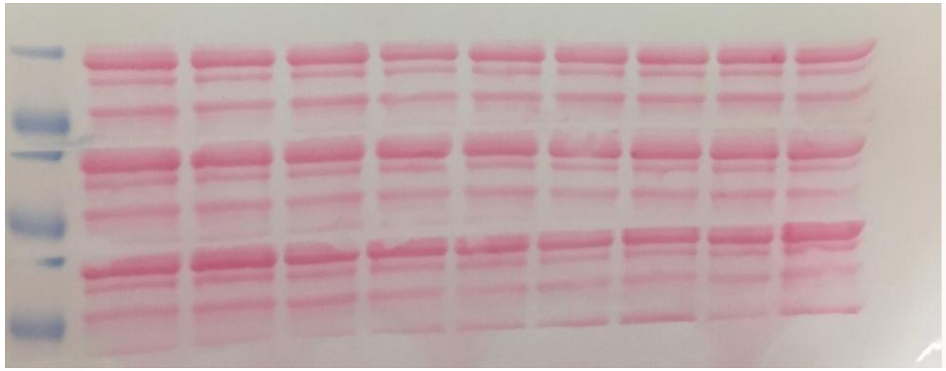

Ponceau

GAPDH

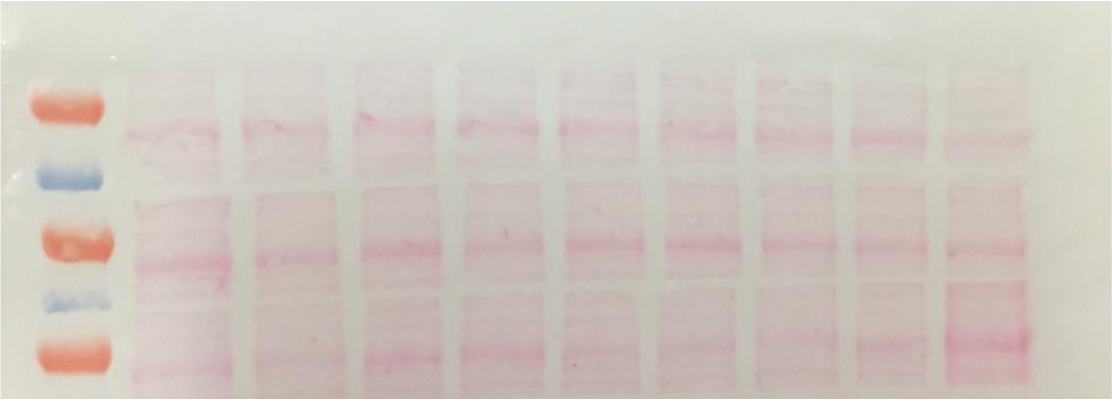

Ponceau

p-70

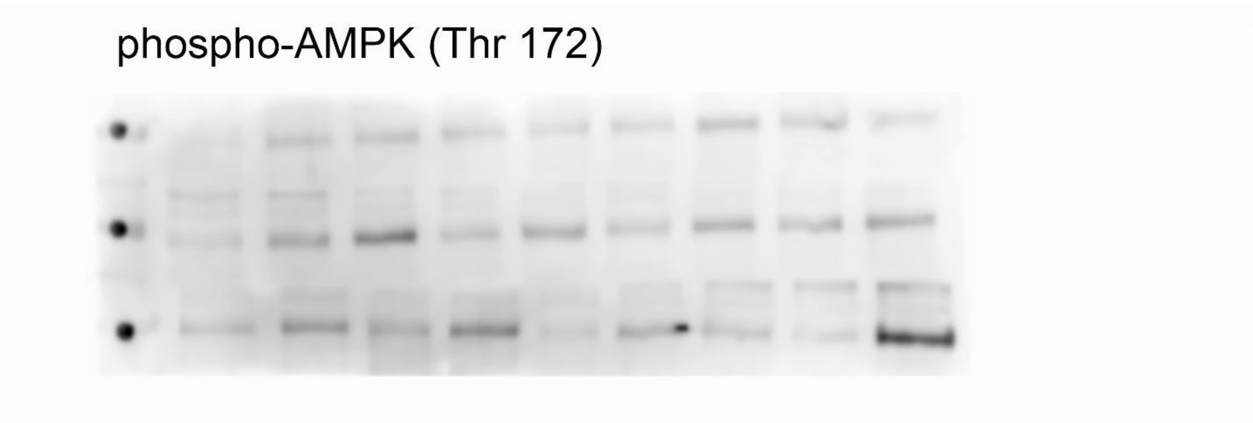
phospho-AMPK (Thr 172)

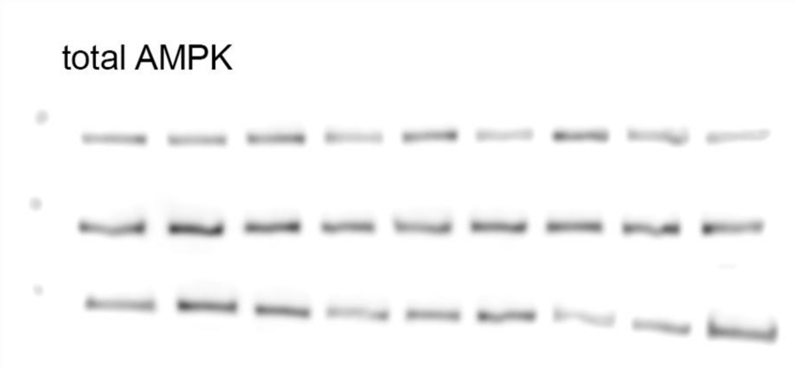
total AMPK

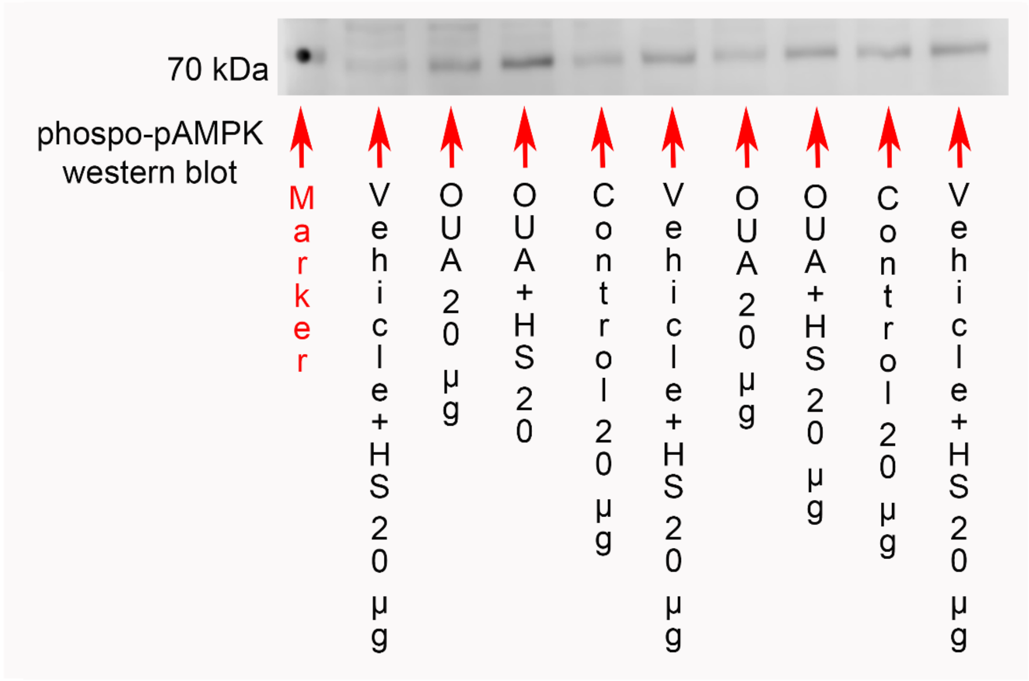
70 kDa
phospo-pAMPK western blot
Marker
Vehicle+HS 20 µg
OUA 20 µg
OUA+HS 20
Control 20 µg
Vehicle+HS 20 µg
OUA 20 µg
OUA+HS 20 µg
Control 20 µg
Vehicle+HS 20 µg

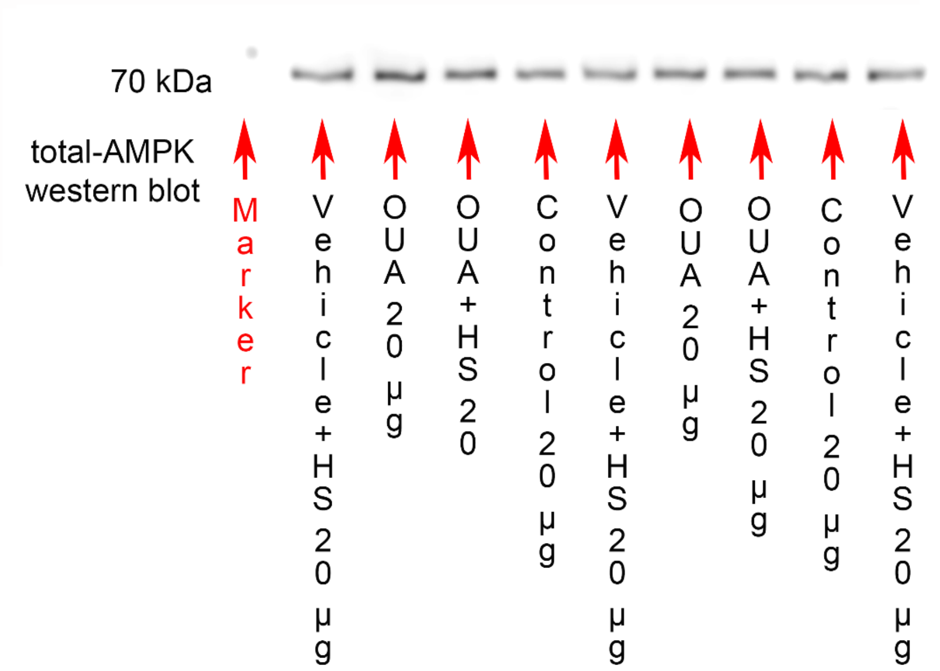
70 kDa
total-AMPK western blot
Marker
Vehicle+HS 20 µg
OUA 20 µg
OUA+HS 20
Control 20 µg
Vehicle+HS 20 µg
OUA 20 µg
OUA+HS 20 µg
Control 20 µg
Vehicle+HS 20 µg

phospho ACC
(Ser 79)

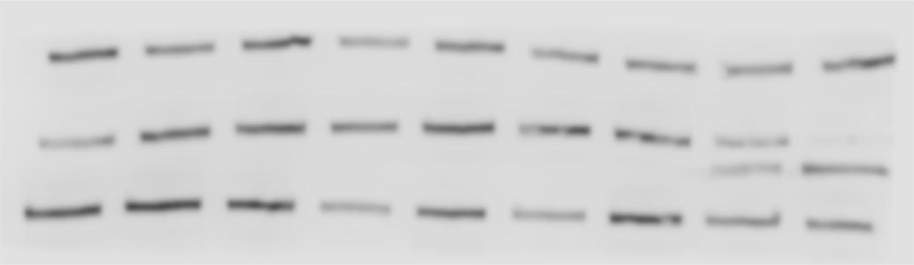

total ACC

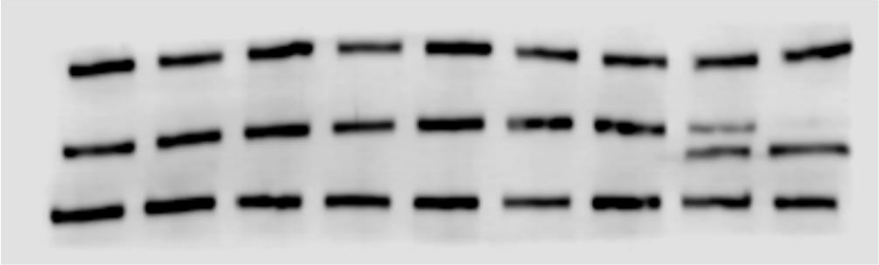

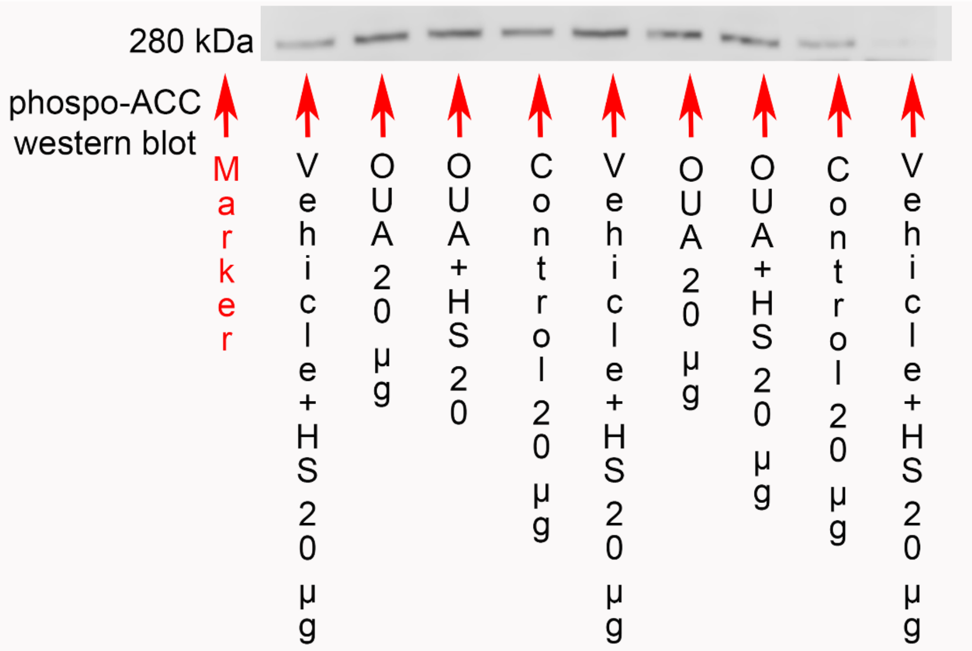

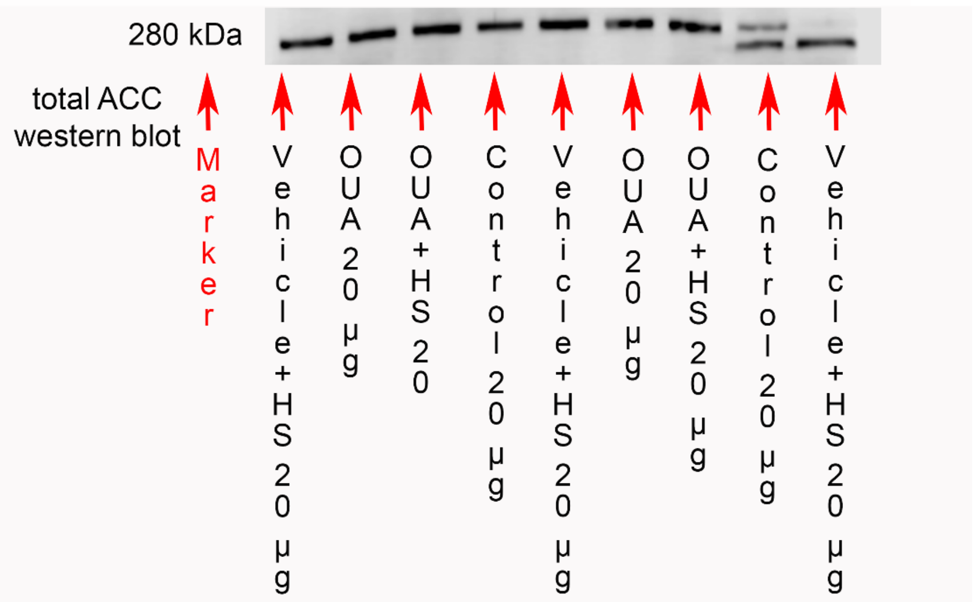

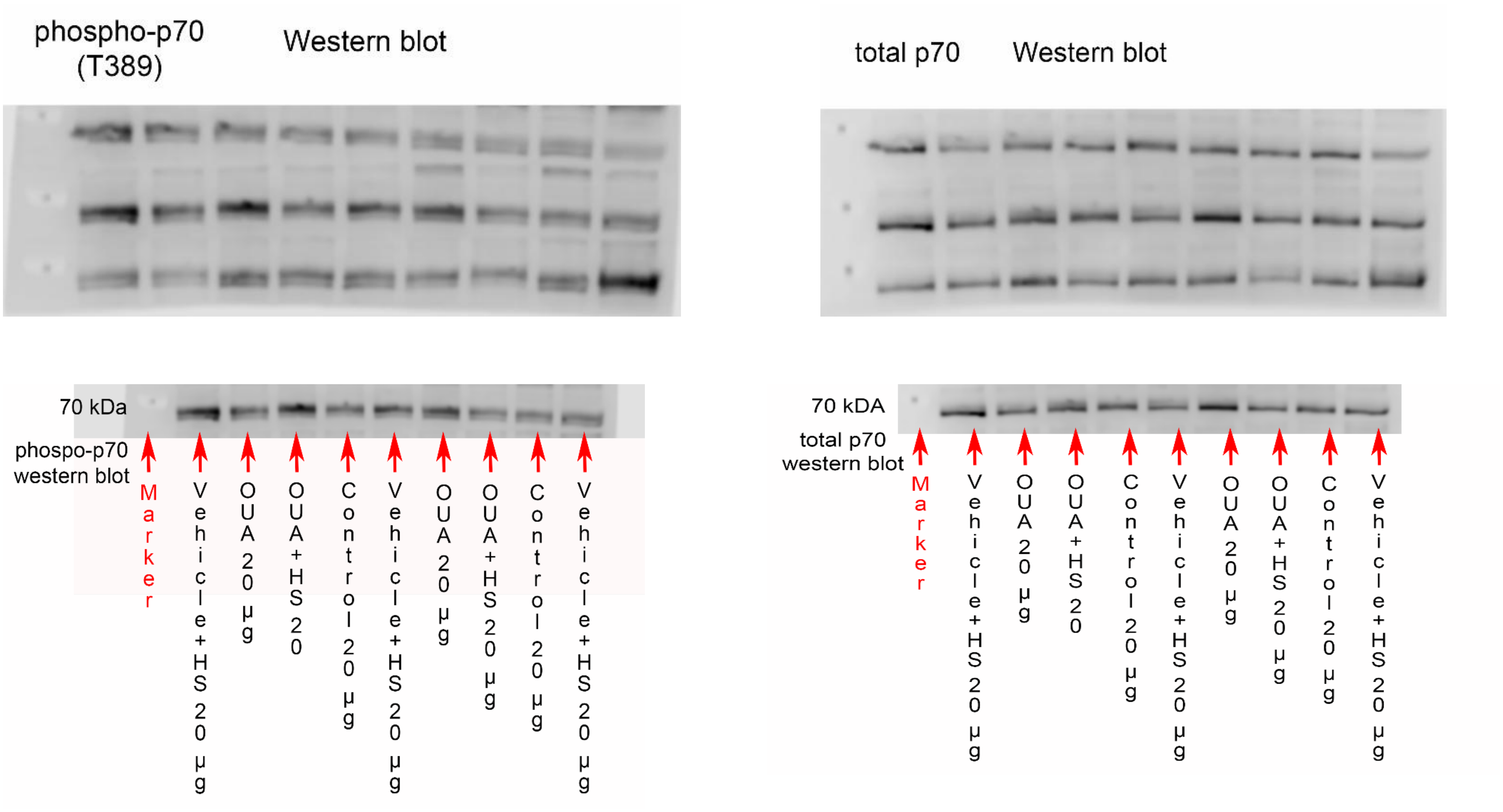
phospho-p70
(T389)
Western blot
70 kDa
phospo-p70
western blot
Marker
Vehicle+HS 20 µg
OUA 20 µg
OUA+HS 20
Control 20 µg
Vehicle+HS 20 µg
OUA 20 µg
OUA+HS 20 µg
Control 20 µg
Vehicle+HS 20 µg
total p70
Western blot
70 kDA
total p70
western blot
Marker
Vehicle+HS 20 µg
OUA 20 µg
OUA+HS 20
Control 20 µg
Vehicle+HS 20 µg
OUA 20 µg
OUA+HS 20 µg
Control 20 µg
Vehicle+HS 20 µg
